# Work-Hardening Induced Tensile Ductility of Bulk Metallic Glasses via High-Pressure Torsion

**DOI:** 10.1038/srep09660

**Published:** 2015-04-23

**Authors:** Soo-Hyun Joo, Dong-Hai Pi, Albertus Deny Heri Setyawan, Hidemi Kato, Milos Janecek, Yong Chan Kim, Sunghak Lee, Hyoung Seop Kim

**Affiliations:** 1 Center for Aerospace Materials, Pohang University of Science and Technology, Pohang 790-784, South Korea; 2 Department of Materials Science and Engineering, Pohang University of Science and Technology, Pohang 790-784, South Korea; 3 Institute for Materials Research, Tohoku University, Sendai 980-8577, Japan; 4 Department of Metal Physics, Charles University, 121 16 Prague 2, Czech Republic; 5 Research Institute of Industrial Science & Technology, Pohang 790-600, South Korea

## Abstract

The mechanical properties of engineering materials are key for ensuring safety and reliability. However, the plastic deformation of BMGs is confined to narrow regions in shear bands, which usually result in limited ductilities and catastrophic failures at low homologous temperatures. The quasi-brittle failure and lack of tensile ductility undercut the potential applications of BMGs. In this report, we present clear tensile ductility in a Zr-based BMG via a high-pressure torsion (HPT) process. Enhanced tensile ductility and work-hardening behavior after the HPT process were investigated, focusing on the microstructure, particularly the changed free volume, which affects deformation mechanisms (i.e., initiation, propagation, and obstruction of shear bands). Our results provide insights into the basic functions of hydrostatic pressure and shear strain in the microstructure and mechanical properties of HPT-processed BMGs.

Numerous studies have highlighted the unique performance and atomic structures of BMGs. However, catastrophic failure at low temperatures^1^,^2^,^3^ continues to restrict their application. Such plastic flow localization is a result of softening effects from thermal or mechanical origins, or a combination of both^4^,^5^,^6^. The unique atomic structure of the amorphous nature of BMGs makes significant differences in compressive and tensile deformation behaviors. BMGs exhibit pressure-dependent yield anisotropy (i.e., tension-compression asymmetry)^7^,^8^,^9^. The deformation and fracture of metallic glasses do not occur along 45° to the loading direction (i.e., the exact maximum shear stress plane in polycrystalline materials) under either tension or compression. Under compressive loads, fracture angles, *θ*_C_, between the compressive axis and the shear plane are smaller than 45° 7,9,10. In contrast, the tensile fracture angles, *θ*_T_, between the loading axis and the shear plane are greater than 45° 7,9,11 because normal stress on the shear plane has a more significant function than shear stress in deformation and fracture. Even though some BMGs show high compressive plasticity, their tensile plasticity is very little.

Over the past decade, several methods have been proposed to increase the ductility of BMGs. Through appropriate composition selection, several BMGs have been found to exhibit reasonably good plasticity^12^,^13^. Moreover, pre-testing of deformation processes, such as rolling^14^,^15^ and shot-peening^16^, can delay catastrophic failure. The results showing increased ductility have been obtained from compression tests. However, significantly increased tensile ductility through pre-treatment has not yet been reported.

Recently, HPT has been developed in order to produce bulk nano/ultrafine structured metallic materials with grain sizes from 20 to 200 nm^17^. Because of its ability to impose extremely high strains compared to other severe plastic deformation (SPD) processes, there have been several studies on HPT-processed BMGs (HPT-BMGs) and amorphous ribbons, as well as polycrystalline materials^18^,^19^,^20^,^21^,^22^. Nevertheless, mechanical property changes due to altered microstructures in HPT-BMGs are not clearly understood.

In the HPT process, applied shear strain (or stress) and hydrostatic pressure ( = −3 × mean normal stress) are two important, independent processing conditions. High pressure and high shear conditions exert a strong influence on free volumes of BMGs. In general, it is supposed that free volume decreases under high pressure^23^,^24^, and that the deformed shear band regions have higher free volume compared to the undeformed matrix^25^,^26^,^27^. Therefore, a systematic understanding of the opposite effects of shear strain and hydrostatic pressure is needed for further improvement of the mechanical properties of BMGs.

In this work, we applied the HPT process (a compression stage followed by torsion under compression) for a tilt-cast Zr_65_Al_7.5_Ni_10_Cu_12.5_Pd_5_ BMG. The mechanical properties of the HPT-BMGs were evaluated using hardness and tensile tests. The deformation behavior of the HPT-BMG was analyzed using a digital image correlation (DIC) method and scanning electron microscopy (SEM). In a nanometer scale, changed free volume and possible nanocrystallization were studied by differential scanning calorimetry (DSC), positron annihilation spectroscopy (PAS), transmission electron microscopy (TEM), and synchrotron x-ray diffraction (XRD). As will be shown in the following report, we suggest that numerous shear band nuclei are formed during tensile tests through shear transformation zones (STZs), which occur preferentially in the softer vacancy cluster regions, and that shear band propagation is impeded by the typical free volume regions and shear band interactions. Furthermore, finite element method (FEM) analysis is performed to theoretically investigate the hydrostatic and shear stress components during HPT, and residual stresses after the HPT process.

## Results

### Enhanced tensile ductility of the HPT-BMGs

**Figure 1:**
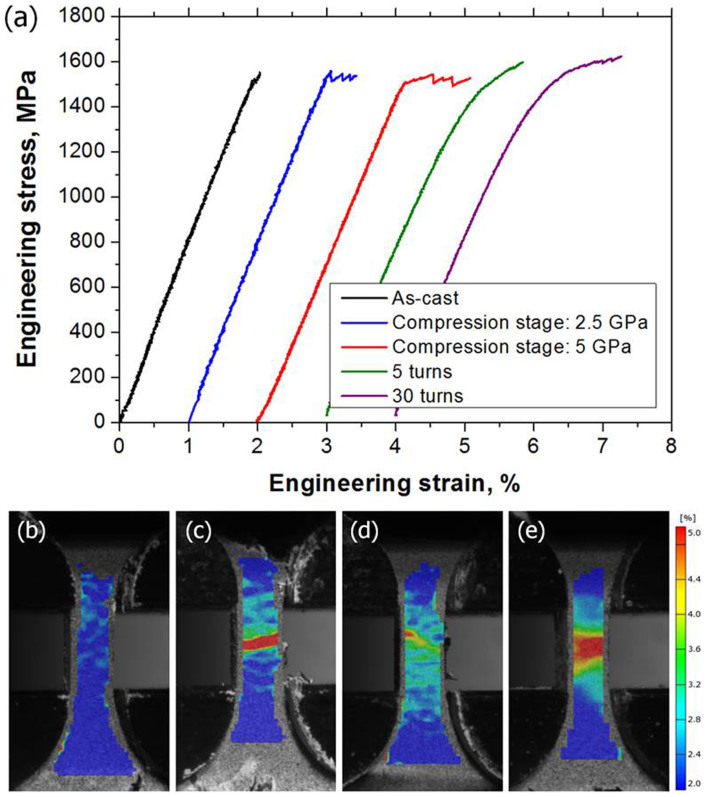
(a) Stress strain curves of the as-cast and HPT-processed BMGs, and strain distribution of (b) the as-cast specimen, (c) the compression-stage HPT specimen, (d) the 5 turn HPT specimen, and (e) the 30 turn HPT specimen before fracture.

Figure 1(a) shows the tensile stress-strain curves of the HPT-BMGs. The measured yield and maximum tensile strengths, fracture strain, and Young's modulus are listed in Table 1. The as-cast specimen presents little plastic strain. However, the HPT-BMGs have noticeably increased tensile ductility. Serration flows with large load drops are observed in the compression-stage specimens. The load serrations are attributed to shear-banding operations in nanoindentation^28^ and compression tests^1^,^2^. The yield strengths of the as-cast and compression-stage HPT specimens are similar. Tensile deformation behaviors of the torsional-stage HPT specimens are different from those of the compression stages. The yield strengths of the torsional-stage HPT specimens are lower than those of the as-cast specimen; however, the stress-strain curves demonstrate clear work-hardening behavior. Eventually, the maximum strengths exceed the yield strength of the as-cast specimen. In the plastic regime, a few small detectable load drops are observed, but the magnitudes of the load drops are much lower than those in the compression-stage HPT specimens.

Figures 1(b–e) shows the strain distributions in the tensile specimens just before (less than 1.5 sec) the fractures were evaluated using the DIC method. The as-cast specimen (Figure 1(b)) shows only elastic deformation. In the compression-stage specimens, highly localized deformation regions are observed as a narrow band (Figure 1(c)). In the 5 turn HPT tensile specimen (Figure 1(d)), the plastic deformation region is larger than that in the compression stage HPT specimen, and a highly deformed region is distinguishable. On the other hand, the strain distribution of the 30 turn specimen (Figure 1(e)) is rather homogeneous. Moreover, strain values are very high (greater than 5%) at some areas. The work-hardening behavior results in homogeneous deformation accompanied by stable necking. The transvers, ε_x_, at the necking region and the other regions are −2.8% and −1.0%, respectively. The necking behavior in the torsional-stage specimens (Figure 1(e)) differs from crystalline metals where the engineering strength decreases after the onset of necking. Moreover, necking occurs by multiple shear banding. Due to the created shear offsets during shear banding, the cross-sectional area is decreased. However, deformation occurs rather homogeneously because the strength of the material is increased by a higher proportion than in the decreased cross-section area. Therefore, the engineering stress gradually increases with strain increment.

### Free volume characterization

**Figure 2:**
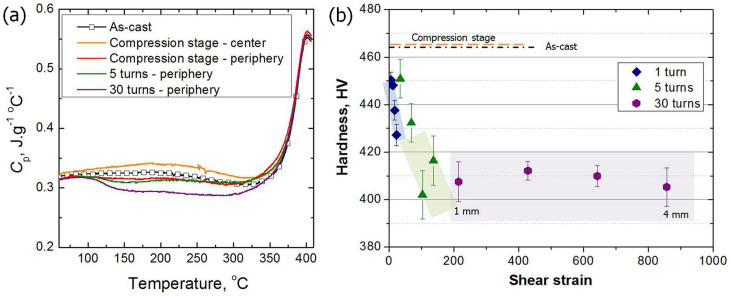
(a) The specific heat, *C*_p_, calculated from the DSC scans, (b) microhardness of the as-cast and HPT-processed BMGs as a function of shear strain.

Microstructural evolutions can be investigated using thermal analysis. In particular, thermal analysis using DSC has been widely used for determining the amount of free volume in BMGs^29^,^30^. Glass transition is considered the result of a specific aspect of structural relaxation, viz. the kinetic process due to the free volume being out of equilibrium and constantly approaching equilibrium during continuous heating in DSC experiments^29^. The specific heat data are shown in Figure 2(a). After the HPT process, all periphery regions exhibit structural relaxation at lower temperatures compared to the as-cast specimen. On the other hand, the center region of the compression stage specimen exhibits increasing specific heat at low temperatures. The enthalpy change upon structural relaxation in BMGs is primarily due to the change in free volume. Accordingly, it can be confirmed that plastic strain in the periphery region increases free volume (disordering) during the HPT process. Furthermore, hydrostatic pressure induces free volume annihilation (ordering). Interestingly, disordering seems to occur from both high and low activation energy sites (high and low temperature sites), as shown in the periphery region of the 30 turn HPT sample.

### Effect of shear strain and hydrostatic pressure

The hardness at the center of the compression stage specimen increased slightly (Figure 2(b)) due to the reduced free volume under hydrostatic pressure. The shear strain, *γ*, generated by simple torsion is proportional to the distance from the center, *r*, as follows:
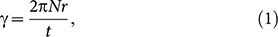
where *N* is the number of HPT turns and *t* is the disk thickness during torsional deformation. Figure 2(b) presents the hardness results as a function of the calculated shear strain, Eq. (1). The hardness of the 1 and 5 turn HPT-BMG specimens decreases along the radial direction, from the center to the periphery. On the other hand, the 30 turn HPT-BMG specimen exhibits significantly decreased hardness all over the sample. Over a shear strain of 200, hardness is minimized to approximately 410 HV, which is 55 HV lower than the as-cast specimen. The decrease in hardness is attributed to free volume generation, i.e., the softening induced by plastic strain.

The hydrostatic stress and shear strain have inverse effects on the microstructure and mechanical properties. The effect of plastic strain on the hardness and the DSC results is more crucial than that of the hydrostatic pressure in the experimental ranges (0 < *γ* < 900, and 0 < P < 5 GPa).

### Multiple shear bands and rugged fracture surface

**Figure 3:**
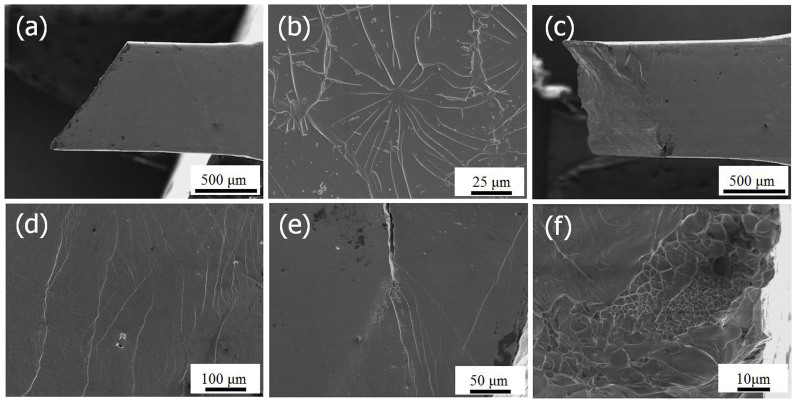
SEM images of (a) lateral surface of the as-cast specimen after a tensile test, (b) a combined vein and core features in the as-cast specimen, (c) lateral surface of the 30 turn specimen after a tensile test, (d) multiple shear bands on the 30 turn specimen, (e) shear band multiplication at the crack tip of the 30 turn specimen, (f) cell-like vein patterns of the 30 turn specimen.

Figure 3(a) is an SEM image of the lateral surface of the fractured as-cast specimen. Even minor shear bands are not detected anywhere. The tensile specimens of the as-cast BMG are fractured straight through the tensile fracture angle, *θ*_T_ = ~55°. In contrast, the fracture angles of the torsional stage specimens do not exhibit a typical tensile fracture angle (Figure 3(c)). The fracture surface of the as-cast specimen has a combined feature of veins and cores (Figure 3(b)). In the 30 turn HPT-BMG specimen, multiple shear bands formed, as seen in Figure 3(d). In addition, a large crack was generated during the tensile test, but crack propagation was stopped by shear band multiplication at the crack tip (Figure 3(e)). This phenomenon implies that the HPT-BMG has good toughness and reliable tensile properties. After the torsional stage HPT, fracture surfaces are rugged as opposed to straight, signifying high fracture resistance. Astonishingly, cell-like vein patterns, instead of cores, are found in the 5 and 30 turn specimens (Figure 3(f)).

### Pre-existing shear bands in the HPT-BMG

**Figure 4:**
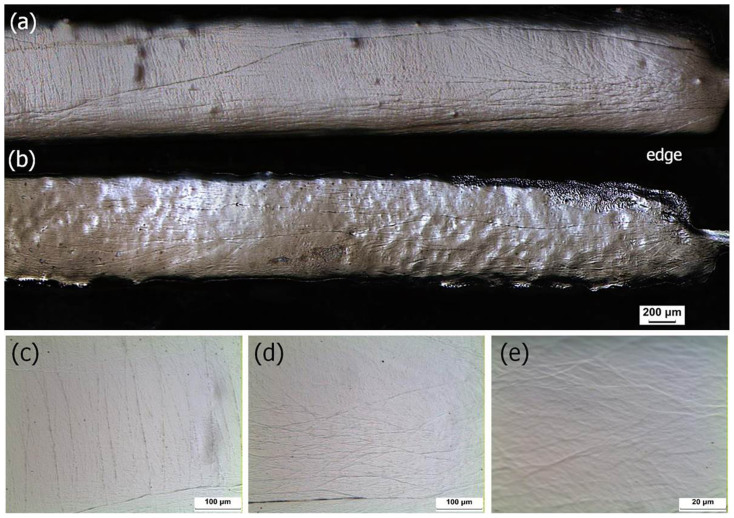
OM images of overall structure of pre-existing shear bands of (a) the 5 turn and (b) 30 turn specimens, (c) the pre-existing shear bands at the center of 5 turn specimen, (d) the wavy pre-existing shear bands at the periphery of 5 turn specimen, and (e) generated minor shear bands of 5 turn specimen.

The observation of shear banding helps us to understand the deformation behavior of BMGs under various stress states. However, the deformation behavior of the BMGs during the HPT process is not yet understood. An understanding of shear band generation and the shapes of shear bands is crucial in the study of the deformation behavior of BMGs during the HPT process. After electro-etching, the as-cast specimen exhibits a flat surface without any noticeable features (not shown here). However, in the 5 and 30 turn HPT-BMG specimens in Figure 4, there are a number of pre-existing shear bands, which make up the preferred etching site. It is verified that shear banding is the primary deformation mechanism of BMGs during the HPT process. In the 5 turn specimen, there are many prominent shear bands through the thickness direction at the disk center (Figures 4(a) and 4(c)). Near the periphery region, wavy shear bands are conspicuous through the radial direction of the disk (Figures 4(a) and 4(d)). In the 30 turn specimen, wavy shear bands are observed on the overall radial surface along the radial direction (Figure 4(b)). The 30 turn HPT-BMG specimen has the lowest hardness at the center compared with the other specimens. Systemic experiments were recently conducted by nanoindentation on shear bands^25^. They revealed that the minimal hardness of a shear band is independent of plastic strain, but that the width of the softening region near a shear band increases with strain. After 30 turns of the HPT process, minimum hardness at the center should be related to the increased density of the shear bands and the enlarged softening region near a shear band. Figure 4(e) is a higher magnification image, demonstrating selectively etched minor shear bands at the periphery region.

The distances between the minor shear bands are less than 20 μm. The formation mechanism of these minor shear bands is unclear, but it should be related to microstructural incompatibilities. Plastic strain during the torsional stage of the HPT process is extremely high, and material flow is not as simple as in uniaxial tests. Therefore, a large number of prominent shear bands are necessary in order to accommodate the high strain in HPTs. However, prominent shear bands are not sufficient to deal with overall deformation because shear banding is a 2D planar motion due to a shear band's small thickness. In order to maintain compatibility during the HPT process, minor shear bands are necessary, such as in geometrically necessary dislocations in polycrystalline materials.

### Evolution of free volume

**Figure 5:**
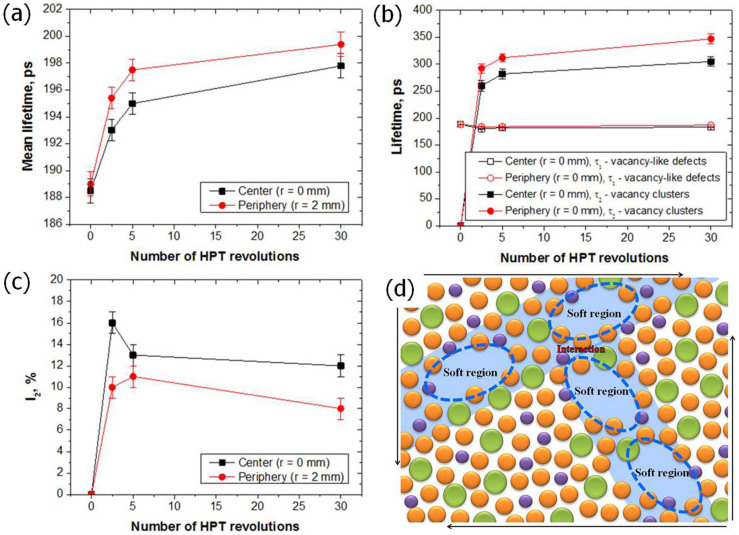
(a) Development of the mean positron lifetime with increasing number of HPT turns, (b) lifetimes of the exponential components resolved in LT spectra, and (c) the relative intensity of the vacancy cluster component, (d) schematic diagram of atomic structure of the HPT processed BMG and multiple shear bands nucleation.

The positron annihilation lifetime measurement results for all specimens are summarized in Figure 5. The development of the mean positron lifetime (τ*_mean_*) with an increase in the number *N* of HPT turns is plotted in Figure 5(a). The τ*_mean_* is a robust parameter that is unaffected by mutual correlations between individual fitting parameters and is able to demonstrate general trends in the specimens. The τ*_mean_* increases with an increase in the number of HPT turns due to new defects introduced during the HPT process. The highest increase of τ*_mean_* can be seen at the beginning of the torsional stage (*N* ≤ 5). Moreover, typically, the τ*_mean_* at the periphery is higher than that in the center.

Decomposition of the positron lifetime spectra reveals that the as-cast BMG specimen exhibits a single component spectrum with a lifetime of ~189 ps. Because the specimens are amorphous, it is difficult to calculate positron lifetimes by ab-initio theoretical calculations. However, as a rough estimation, the weighted average of the positron lifetime of the pure elements that constitute the BMG can be used. This estimation yields a free positron lifetime of ~140 ps and a lifetime of ~220 ps for positrons trapped at vacancies. Hence, a lifetime of 189 ps can be attributed to the positrons trapped at vacancy-like defects (free volume) already existing in the as-cast specimen.

An additional component with lifetime τ*_2_* > 250 ps was detected in the HPT-BMG specimens. This is obviously the contribution of positrons trapped at larger point defects with open volumes comparable to a cluster of several vacancies. The development of positron lifetimes by increasing the number of HPT turns is plotted in Figure 5(b), while Figure 5(c) exhibits the relative intensity of the vacancy cluster component. Figures 5(b) and 5(c) show that the lifetime τ*_1_* corresponding to positrons trapped at vacancy-like defects is approximately the same in the center as at the periphery, and remains almost unchanged during the HPT process. This verifies that the nature of vacancy-like defects does not change during the HPT process. On the other hand, the lifetime τ*_2_* increases with the number of HPT turns, indicating that the size of vacancy clusters increases during the HPT process. Moreover, the size of vacancy clusters is larger at the periphery because this region is subjected to higher strain. The relative intensity *I_2_* slightly decreases during the HPT process, most probably because the density of vacancy-like defects increases faster than the concentration of vacancy clusters. Note that vacancy-like defects and vacancy clusters are competitive traps. Because of saturated trapping, a positron is trapped either in a vacancy-like defect or in a vacancy cluster. Figure 5(c) shows that the relative intensity *I_2_* is slightly lower at the periphery due to the higher density of vacancy-like defects compared to the center.

### Phase transformation behavior

**Figure 6:**
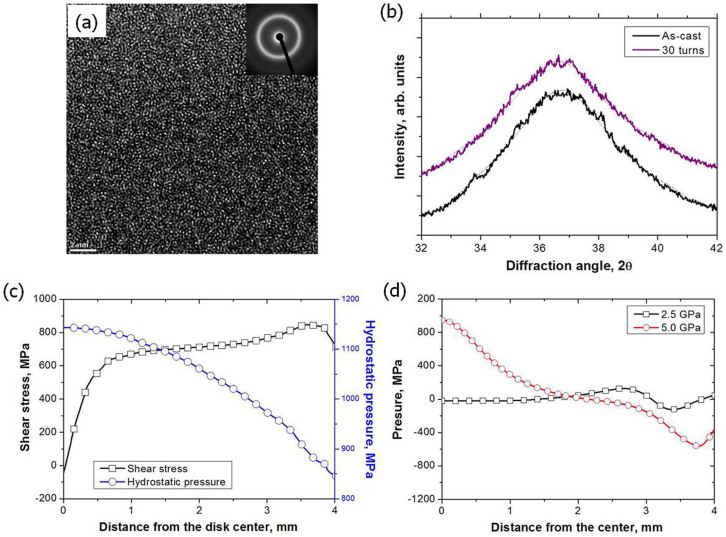
(a) High resolution TEM image of the 30 turn HPT specimen, (b) synchrotron X-ray diffraction patterns, and FEM results on (c) distribution of shear stress and hydrostatic pressure after the 1 turn HPT process under 5 GPa and (d) residual stresses after 2.5 GPa and 5 GPa compression stages along the radial distance from the disk center.

The transformation behavior of BMGs influences their mechanical properties. Therefore, crystallization and nanocrystallization of BMGs have been widely studied^31^,^32^,^33^,^34^. In the present study, possible nanocrystallization was studied using high resolution TEM and synchrotron XRD. After 30 turns of the HPT process, the uniform amorphous structure was not changed due to large plastic deformation (Figure 6(a)). The selected area electron diffraction (SAED) pattern, inserted at the upper right corner, indicates the conservation of an overall fully amorphous structure. There are no fringe contrasts indicating a crystalline structure and no ordered clusters within the glassy structure. In the synchrotron XRD results, which represent a full specimen, significant diffraction peaks were not observed in all specimens (Figure 6(b)). The tiny peaks were unclearly found in the 30 turn HPT-BMG specimens. Therefore, it is concluded that significant nanocrystallization did not occur in the Zr_65_Al_7.5_Ni_10_Cu_12.5_Pd_5_ alloy.

### FEM results and effect of residual stresses

The shear stress and hydrostatic pressure after 1 turn of the 5 GPa HPT process were investigated using the FEM (Figure 6(c)). At the center of the torsion specimen, shear stress is negligible, but hydrostatic pressure is higher. Shear banding directions can be altered in different stress states. Therefore, in the 5 turn HPT specimen, the direction of shear bands at the center and near the periphery region (in Figure 4(a)) should be different.

Figure 6(d) exhibits residual stresses after the 2.5 GPa and 5 GPa compression stages. After the 2.5 GPa compression stage, the residual stress is negligible at the center. However, after the 5 GPa compression stage, the residual pressure ( = −3 × mean stress) is over 800 MPa. It is known that compressive residual stress induced by shot peening enhances compressive ductility^16^. The increased tensile ductility values of the 2.5 GPa and 5 GPa compression stage specimens were 0.47% and 0.97%, respectively. Therefore, compressive residual stresses after the compression stage of the HPT process prevent propagation of shear banding.

## Discussion

The aim of our approach is to increase tensile ductility in monotonic BMGs and to understand the mechanisms of work-hardenability. Tensile ductility is rarely observed in monotonic BMGs, thus the HPT process can provide guidance for how to develop BMGs as reliable engineering materials.

Our results revealed the HPT-BMGs have heterogeneous microstructures, which are a mixture of severely deformed shear bands and undeformed matrixes. The microstructural heterogeneity prevents strain localization. Local yielding of the HPT-BMG should possibly occur at shear bands that were generated along a direction similar to the typical tensile fracture angle. However, propagation of the tension-induced shear bands is prohibited by the neighboring pre-existing shear bands and undeformed matrix. The prohibited shear bands do not propagate further, and then the other shear bands having different propagation directions are newly initiated at a higher stress. These initiation, propagation, and obstruction processes of the shear bands are repeated during the tensile test of the HPT-BMGs and are represented as the work-hardening phenomenon in stress-strain curves. Even though the tensile specimens are uniaxially loaded, the HPT-BMG is locally deformed in a complex deformation mode, as opposed to a uniaxial mode. These deformation mechanisms are similar to BMG composites^35^. Therefore, complicated rugged fracture surfaces with cell-like vein patterns are created and cores are not formed on the fracture surfaces.

Moreover, the wavy and tangled pre-existing shear bands are more beneficial for tensile ductility than the pre-existing shear bands, which align along the thickness direction of tensile specimens. The HPT process has the advantage of imposing huge strain and suppressing cracks and pores. Ultimately, work-hardening behavior with substantial tensile ductility results from the multiple shear banding caused by uniformly distributed heterogeneous microstructures without cracks or pores after 30 turns of the HPT process.

Our results also reveal that the HPT process generates vacancy-cluster sizes of new free volume. The shear transformation zones (STZs) are the elementary mechanism of inhomogeneous deformation in metallic glasses. The STZs serve as nucleation sites for shear bands, and the formation of STZs are dependent on the local microstructure^12^. In a nanometer scale, the microstructure of the HPT-BMG is heterogeneous (Figure 5(d)). Soft regions include vacancy clusters, and typical free volume regions can be defined as hard regions. The STZs should occur preferentially in the vacancy cluster regions and numerous shear band nuclei are formed along the soft regions. However, the hard regions impede shear band propagation and change the shear band propagation directions. Also, shear band interactions can block their propagation^2^. Theses obstructions contribute to shear band multiplications, which results in superior plasticity^36^. The higher turns of the HPT process increase the tensile ductility of the BMG and the size of the vacancy clusters. It is worth noting that the vacancy clusters do not act like brittle crack initiate sites.

We note that developed tensile ductility of the HPT-BMG does not result from nanocrystallization. Although the mechanism for deformation induced crystallization is still under discussion, recent research indicates that segregation and transformation are induced by local temperature increase or by a change in the chemical short-range order (CSRO), which are caused by the concentration of shear stress^37^. The phase transformation behavior becomes more complicated when hydrostatic pressure is applied like in the HPT process. The hydrostatic pressure may influence the thermodynamic potential energy barrier of nucleation. The nucleation rate (*I*) is expressed by
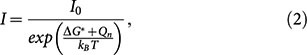
where *I*_0_ is a constant factor, *k_B_* the Boltzmann's constant, *Q*_n_ the activation energy of transport of an atom across interface of an embryo, and G* the free energy required to form a nucleus of the critical size. In general, the hydrostatic pressure could reduce atomic mobility, therefore, *Q*_n_ increases with increasing *P*. According to Jiang et al.^34^, ΔG* + *Q*_n_, which is the so-called nucleation work, could be influenced by applied external pressure. ΔG* is proportional to *σ*^3^/(*p*Δ*V* + Δ*G*)^2^, where *σ*, Δ*V*, and Δ*G* are interfacial energy of the crystal, change of molar volume at activation or final crystalline states, and change of free energy. Therefore, the sign of Δ*V* determines the hydrostatic pressure effect on the nucleation barrier^33^.

Recently, the nanocrystallization of some metallic glasses after the HPT process has been reported^20^,^21^,^22^,^38^,^39^,^40^,^41^. However, other reports have not shown any phase transformation during the HPT process^18^,^19^,^42^,^43^. This unclear tendency is also observed during indentation tests of various BMGs^44^,^45^. When nanocrystallization occurs, the propensity of nanocrystallization is much more prominent near the indent region compared to that of the regions far from the indent^45^. Therefore, depending on the chemistry and processing (strain rate or hydrostatic pressure) the nanocrystllization may not occur. The Zr_65_Al_7.5_Ni_10_Cu_12.5_Pd_5_ shows deformation-induced nanocrystallization during compression^46^, but we cannot observe nanocrystalline phases after the HPT process. Furthermore, we could detect a single icosahedral phase as the primary phase of both the as-cast and HPT-BMG after annealing at various conditions (Figure S6). These results also imply that nanocrystals do not exist in the HPT-BMG. Eventually, the transformation of the BMGs during the HPT process should be greatly influenced by difference in volume change by the primary crystallization and reduced atomic mobility in high hydrostatic pressure. Nanocrystallization can increase hardness of the HPT-processed BMGs^20^,^40^. However, in this study, hardness of the Zr_65_Al_7.5_Ni_10_Cu_12.5_Pd_5_ alloy decreased dramatically after the torsional-stage HPT because any nanocrystallization did not occur during the HPT process. Here, we suggest that proper free volume changes can enhance the ductilities of monotonic BMGs without second phases or nanocrystallization.

In summary, we observed enhanced tensile ductility and work-hardening behavior in HPT-BMGs. Plastic strain changes free volume more dominantly than hydrostatic pressure. The introduced larger size (vacancy clusters) of new free volumes generates the heterogeneous structure of the BMG in the nanometer scale and causes the multiplication of shear bands. Microstructural heterogeneity prevents strain localization through a single shear band. Thus, strain is concentrated at only small regions, and other shear bands having different propagation directions are newly initiated at a higher stress. The HPT-BMG is locally deformed in a complex deformation mode during tensile tests. Therefore, complicated rugged fracture surfaces with cell-like vein patterns are created, and the cores are not formed anywhere on the fracture surfaces. The high resolution TEM and synchrotron XRD results support that nanocrystallization did not occur even after 30 turns. The compressive residual stresses remained after the compression stage should have prevented the propagation of shear bands.

## Methods

### BMG alloy production

A Zr_55_Al_10_Ni_5_Cu_30_Pd BMG was produced via arc-melting the pure elements (99.99%) under a purified argon atmosphere in button-shaped ingots of the desired composition. The BMG cylindrical rods with a diameter of 8 mm were prepared using tilt-casting into copper molds. Disk shaped preforms for the HPT process were cut into 1.5 mm thickness from the cast rods and then polished to remove possible microstructural changes and surface defects during the cutting.

### HPT process

In the compression stage, the speed of the lower anvil was 0.1 mm·s^−1^ until a pressure of 5 GPa was developed on the workpiece. In the torsional stage, the lower anvil was rotated at 0.1 rad·s^−1^.

### Mechanical testing

Vickers microhardness was estimated in the centerline of the cross-section of the specimen along the radial direction on a radial surface using a Future-Tech FM-700 tester. The applied load and dwell time were 300 g and 10 sec, respectively. For tensile testing, one dog bone type specimen was cut from the BMG disk along a radial direction using a wire cutting machine. A gauge length was 1.25 mm and width was 1.0 mm. The thicknesses of tensile specimens were made to be 0.7 mm for all the conditions. Also, the surfaces of the tensile specimens were fine polished using 1 μm diamond powders and electro-etching to remove surface defects. The tensile tests were performed at room temperature with an initial strain rate of 1.0 × 10^−4^ s^−1^. During the tests, precise strains were measured using the vision strain gauge system (ARAMIS 5M, GOM mbH, Germany) which detects the local 3D coordinates of the full-scale deforming object surface on the basis of digital image processing (DIC)^2^,^47^, delivering also the 3D displacement and strain. More than three tensile tests for each condition were conducted in order to obtain reliable tensile properties. The average speckle size and spacing were both approximately 35 μm, and the facet size (the size of a calculate unit) was 92 μm.

### Microstructure observation

Morphology of the pre-existing shear bands on a radial surface was observed using OM after electro-polishing in 25% nital etchants at 7 V for 30 seconds. Synchrotron XRD measurements were performed to confirm nanocrystallization; the Pohang Steel and Iron Corporation (POSCO) Beamline (8D) at the Pohang Light Source (PLS) in Pohang, Korea was used. Thermal analysis was performed in DSC using a Perkin Elmer DSC 8500 in a purified argon atmosphere. The specimen geometry was 2 mm in length, 1 mm in width, and 0.4 mm in thickness. The specimens, sealed in Al pan, were scanned from 50 to 408°C at a heating rate of 20°C/min. Calculations of a heat capacity at constant pressure, *C*_p_, were calibrated by using a standard sapphire. The TEM specimens were prepared using focused ion beam (FIB, a Helios Nanolab Dual FIB). High resolution TEM images of the BMG were taken using a JEOL JEM-2200FS with an image Cs-corrector. For the positron lifetime measurement, a ^22^Na_2_CO_3_ positron source with the activity of 1.5 MBq deposited on a 2 μm thick Mylar foil was used. The source spot diameter was 1 mm. The source contribution consists of two components with lifetimes of 368 ps (intensity 8%) and 1.5 ns (intensity 1%) which come from positrons annihilated in the ^22^Na_2_CO_3_ source spot and in the covering Mylar foil, respectively. The source component was always subtracted from the spectrum. A fast–fast spectrometer with the time resolution of 150 ps (FWHM ^22^Na) was employed for positron lifetime measurements. At least, 10^7^ positron annihilation events were accumulated in each positron lifetime spectrum which was subsequently decomposed into individual exponential components by a maximum likelihood procedure^48^.

### Finite element simulation

A commercial elasto-plastic analysis FEM software package, ABAQUS, was used to analyze residual stresses. Approximately 14,400 elements were generated to represent the workpiece. An axisymmetric calculation was applied, which was adequate to simulate the plastic deformation and the interaction effect of the HPT disk. The torsional stage of the HPT process was analyzed using DEFORM 3D. Approximately 150,000 elements were used for the torsional stage analysis. The coefficient of friction between the anvils and the workpiece was assumed to be 0.8. The anvils were modeled as rigid non-deformable parts. The BMG was modeled as a perfect plastic material. Young's modulus, yield strength, and Poisson's ratio were defined as 80 GPa, 1,600 MPa, and 0.35, respectively.

## Supplementary Material

Supplementary Information Supplementary Information

## Figures and Tables

**Table 1:** Mechanical properties obtained from the tensile tests of the as-cast and HPT-processed BMG

	Yield strength (MPa)	Maximum strength (MPa)	Fracture strain (%)	Young's modulus (GPa)
As-cast	1542 ± 27	1545 ± 24	2.06 ± 0.05	69.9 ± 2.8
Compression stage (2.5 GPa)	1547 ± 8	1555 ± 14	2.47 ± 0.27	72.2 ± 2.6
Compression stage (5 GPa)	1537 ± 13	1539 ± 12	2.97 ± 0.15	73.8 ± 0.7
5 turns	1216 ± 28	1622 ± 29	2.77 ± 0.05	69.8 ± 1.5
30 turns	1118 ± 15	1642 ± 16	3.43 ± 0.32	68.9 ± 1.5

## References

[b1] Song S. X., Bei H., Wadsworth J. & Nieh T. G. Flow serration in a Zr-based bulk metallic glass in compression at low strain rates. Intermetallics 16, 813–818 (2008).

[b2] Joo S.-H., Kato H., Gangwar K., Lee S. & Kim H. S. Shear banding behavior and fracture mechanisms of Zr_55_Al_10_Ni_5_Cu_30_ bulk metallic glass in uniaxial compression analyzed using a digital image correlation method. Intermetallics 32, 21–29 (2013).

[b3] Mukai T. *et al.* Effect of strain rate on compressive behavior of a Pd_40_Ni_40_P_20_ bulk metallic glass. Intermetallics 10, 1071–1077 (2002).

[b4] Cheng Y., Han Z., Li Y. & Ma E. Cold versus hot shear banding in bulk metallic glass. Phys. Rev. B 80, 134115 (2009).

[b5] Lewandowski J. J. & Greer A. L. Temperature rise at shear bands in metallic glasses. Nat. Mater. 5, 15–18 (2005).

[b6] Wright W. J., Schwarz R. B. & Nix W. D. Localized heating during serrated plastic flow in bulk metallic glasses. Mater. Sci. Eng. A 319–321, 229–232 (2001).

[b7] Zhang Z., He G., Eckert J. & Schultz L. Fracture Mechanisms in Bulk Metallic Glassy Materials. Phys. Rev. Lett. 91, 045505 (2003). 12906675 10.1103/PhysRevLett.91.045505

[b8] Lowhaphandu P., Montgomery S. L. & Lewandowski J. J. Effects of superimposed hydrostatic pressure on flow and fracture of a Zr-Ti-Ni-Cu-Be bulk amorphous alloy. Scripta Mater. 41, 19–24 (1999).

[b9] Zhang Z. F., Eckert J. & Schultz L. Difference in compressive and tensile fracture mechanisms of Zr_59_Cu_20_Al_10_Ni_8_Ti_3_ bulk metallic glass. Acta Mater. 51, 1167–1179 (2003).

[b10] Wright W. J., Saha R. & Nix W. D. Deformation mechanisms of the Zr_40_Ti_14_Ni_10_Cu_12_Be_24_ bulk metallic glass. Mater. Trans. 42, 642–649 (2001).

[b11] Mukai T. *et al.* Dynamic response of a Pd_40_Ni_40_P_20_ bulk metallic glass in tension. Scripta Mater. 46, 43–47 (2002).

[b12] Liu Y. H. *et al.* Super plastic bulk metallic glasses at room temperature. Science 315, 1385–1388 (2007). 17347434 10.1126/science.1136726

[b13] Zhang L. *et al.* Bulk metallic glasses with large plasticity: Composition design from the structural perspective. Acta Mater. 57, 1154–1164 (2009).

[b14] Kobata J. *et al.* Effect of Pre-Introduced Shear Bands Direction on Deformation Behavior in Zr_55_Al_10_Ni_5_Cu_30_ Bulk Metallic Glass. Mater. Trans. 50, 2355–2358 (2009).

[b15] Lee M. H. *et al.* Improved plasticity of bulk metallic glasses upon cold rolling. Scripta Mater. 62, 678–681 (2010).

[b16] Zhang Y., Wang W. H. & Greer A. L. Making metallic glasses plastic by control of residual stress. Nat. Mater. 5, 857–860 (2006). 17041581 10.1038/nmat1758

[b17] Zhilyaev A. & Langdon T. Using high-pressure torsion for metal processing: Fundamentals and applications. Prog. Mater. Sci. 53, 893–979 (2008).

[b18] Révész A. D. M., Schafler E. & Kovács Z. Structural anisotropy in a Zr_57_Ti_5_Cu _20_Al_10_Ni_8_ bulk metallic glass deformed by high pressure torsion at room temperature. Appl. Phys. Lett. 92, 011910 (2008).

[b19] Wang X. D. *et al.* Atomic-level structural modifications induced by severe plastic shear deformation in bulk metallic glasses. Scripta Mater. 64, 81–84 (2011).

[b20] Wang Y. B. *et al.* Introducing a strain-hardening capability to improve the ductility of bulk metallic glasses via severe plastic deformation. Acta Mater. 60, 253–260 (2012).

[b21] Hóbor S. N. *et al.* High pressure torsion of amorphous Cu_60_Zr_30_Ti_10_ alloy. J. Appl. Phys. 104, 033525 (2008).

[b22] Van Steenberge N. *et al.* Effects of severe plastic deformation on the structure and thermo-mechanical properties of Zr_55_Cu_30_Al_10_Ni_5_ bulk metallic glass. J. Alloys Comp. 500, 61–67 (2010).

[b23] Jiang J. Z. *et al.* Pressure effect on crystallization of metallic glass Fe_72_P_11_C_6_Al_5_B_4_Ga_2_ alloy with wide supercooled liquid region. J. Appl. Phys. 87, 2664–2666 (2000).

[b24] Wang W. H. *et al.* Nanocrystallization of ZrTiCuNiBeC bulk metallic glass under high pressure. Appl. Phys. Lett. 75, 2770–2772 (1999).

[b25] Pan J., Chen Q., Liu L. & Li Y. Softening and dilatation in a single shear band. Acta Mater. 59, 5146–5158 (2011).

[b26] Wright W. J., Hufnagel T. C. & Nix W. D. Free volume coalescence and void formation in shear bands in metallic glass. J. Appl. Phys. 93, 1432–1437 (2003).

[b27] Steif P. S., Spaepen F. & Hutchinson J. W. Strain localization in amorphous metals. Acta Metall. 30, 447–455 (1982).

[b28] Schuh C. A. & Nieh T. G. A nanoindentation study of serrated flow in bulk metallic glasses. Acta Mater. 51, 87–99 (2003).

[b29] Van den Beukel A. & Sietsma J. The glass transition as a free volume related kinetic phenomenon. Acta Metall. Mater. 38, 383–389 (1990).

[b30] Slipenyuk A. & Eckert J. Correlation between enthalpy change and free volume reduction during structural relaxation of Zr_55_Cu_30_Al_10_Ni_5_ metallic glass. Scripta Mater. 50, 39–44 (2004).

[b31] Lee J.-C. *et al.* Deformation-induced nanocrystallization and its influence on work hardening in a bulk amorphous matrix composite. Acta Mater. 52, 1525–1533 (2004).

[b32] Saida J., Setyawan A. D. H., Kato H. & Inoue A. Nanoscale multistep shear band formation by deformation-induced nanocrystallization in Zr-Al-Ni-Pd bulk metallic glass. Appl. Phys. Lett. 87, 151907 (2005).

[b33] Kato H., Saida J. & Inoue A. Influence of hydrostatic pressure during casting on as cast structure and mechanical properties in Zr65Al7.5Ni10Cu17.5−xPdx (x = 0, 17.5) alloys. Scripta Mater. 51, 1063–1068 (2004).

[b34] Jiang J. Z., Gerward L. & Xu Y. S. Pressure effect on crystallization kinetics in Zr_46.8_Ti_8.2_Cu_7.5_Ni_10_Be_27.5_ bulk glass. Appl. Phys. Lett. 81, 4347–4349 (2002).

[b35] Jeon C. *et al.* High tensile ductility of Ti-based amorphous matrix composites modified from conventional Ti–6Al–4V titanium alloy. Acta Mater. 61, 3012–3026 (2013).

[b36] Chen M. Mechanical behavior of metallic glasses: Microscopic understanding of strength and ductility. Annu. Rev. Mater. Res. 38, 445–469 (2008).

[b37] Chen H., He Y., Shiflet G. J. & Poon S. J. Deformation-induced nanocrystal formation in shear bands of amorphous alloys. Nature 367, 541–543 (1994).

[b38] Henits P., Révész Á., Varga L. K. & Kovács Z. The evolution of the microstructure in amorphous Al_85_Ce_8_Ni_5_Co_2_ alloy during heat treatment and severe plastic deformation: A comparative study. Intermetallics 19, 267–275 (2011).

[b39] Henits P. *et al.* Nanocrystallization in Al_85_Ce_8_Ni_5_Co_2_ amorphous alloy obtained by different strain rate during high pressure torsion. J. Alloys Comp. 504, S91–S94 (2010).

[b40] Kovács Z. *et al.* Stability of medium range order in Al-based metallic glass compacted by severe plastic deformation. J. Alloys Comp. 561, 5–9 (2013).

[b41] Sort J. *et al.* Cold-consolidation of ball-milled Fe-based amorphous ribbons by high pressure torsion. Scripta Mater. 50, 1221–1225 (2004).

[b42] Révész Á., Henits P. & Kovács Z. Structural changes in Zr-based bulk metallic glasses deformed by high pressure torsion. J. Alloys Comp. 495, 338–340 (2010).

[b43] Meng F., Tsuchiya K., Seiichiro & Yokoyama Y. Reversible transition of deformation mode by structural rejuvenation and relaxation in bulk metallic glass. Appl. Phys. Lett. 101, 121914 (2012).

[b44] Mukhopadhyay N. K., Belger A., Paufler P. & Kim D. H. Nanoindentation studies on Cu-Ti-Zr-Ni-Si-Sn bulk metallic glasses. Mater. Sci. Eng. A 449-451, 954–957 (2007).

[b45] Mukhopadhyay N. K. & Paufler P. Micro- and nanoindentation techniques for mechanical characterisation of materials. Int. Mater. Rev. 51, 209–245 (2006).

[b46] Saida J., Setyawan A. D., Kato H., Matsushita M. & Inoue A. Improvement of plasticity in Pd containing Zr-Al-Ni-Cu bulk metallic glass by deformation-induced nano structure change. Mater. Trans. 49, 2732–2736 (2008).

[b47] Joo S.-H. *et al.* Method for measuring nanoscale local strain in a dual phase steel using digital image correlation with nanodot patterns. Scripta Mater. 68, 245–248 (2013).

[b48] Procházka I., Novotný I. & Bečvář F. Application of maximum-likelihood method to decomposition of positron-lifetime spectra to finite number of components. Mater. Sci. Forum 255-257, 772–774 (1997).

