# Supplementary material for: Work-Hardening Induced Tensile Ductility of Bulk Metallic Glasses via High-Pressure Torsion
Source: Sci Rep. 2015 Apr 23;5:9660. doi: 10.1038/srep09660 (PMC5386117; doi:10.1038/srep09660)
Supplement: Supplementary Information [file srep09660-s1.pdf]

Supporting Information

Work-Hardening Induced Tensile Ductility of Bulk Metallic Glasses via High-Pressure Torsion

Soo-Hyun Joo[a], Dong-Hai Pi[b], Albertus Deny Heri Setyawan[c], Hidemi Kato[c], Milos Janecek[d], Yong Chan Kim[e], Sunghak Lee[a,b] and Hyoung Seop Kim[a,b,]*

[a]Center for Aerospace Materials, Pohang University of Science and Technology, Pohang 790-784, South Korea

[b]Department of Materials Science and Engineering, Pohang University of Science and Technology, Pohang 790-784, South Korea

[c]Institute for Materials Research, Tohoku University, Sendai 980-8577, Japan

[d]Department of Metal Physics, Charles University, 121 16 Prague 2, Czech Republic

[e]Research Institute of Industrial Science & Technology, Pohang 790-600, South Korea

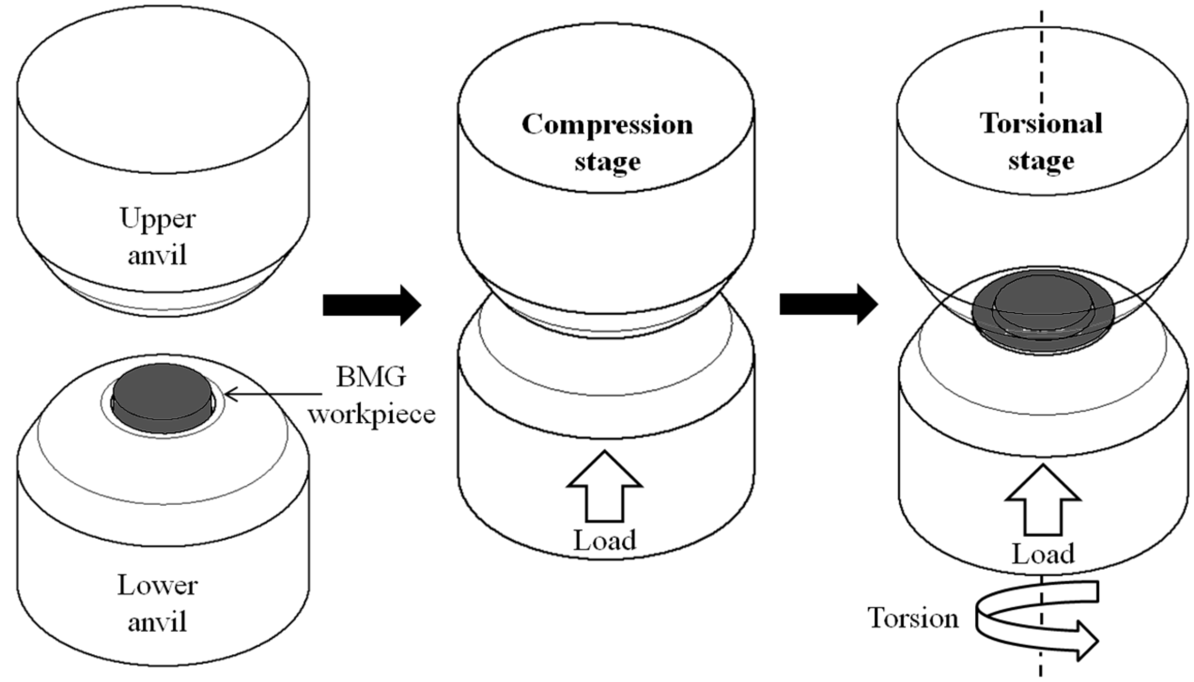

Figure S1. Schematic of the high-pressure torsion process.

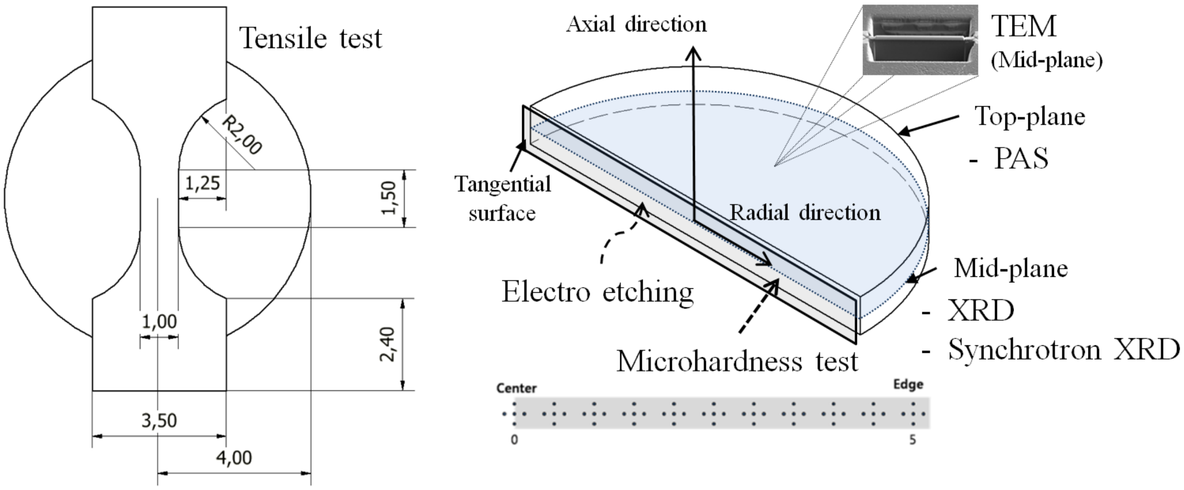

Figure S2. Schematic of performed experiments.

XRD results and HPT-processed specimens

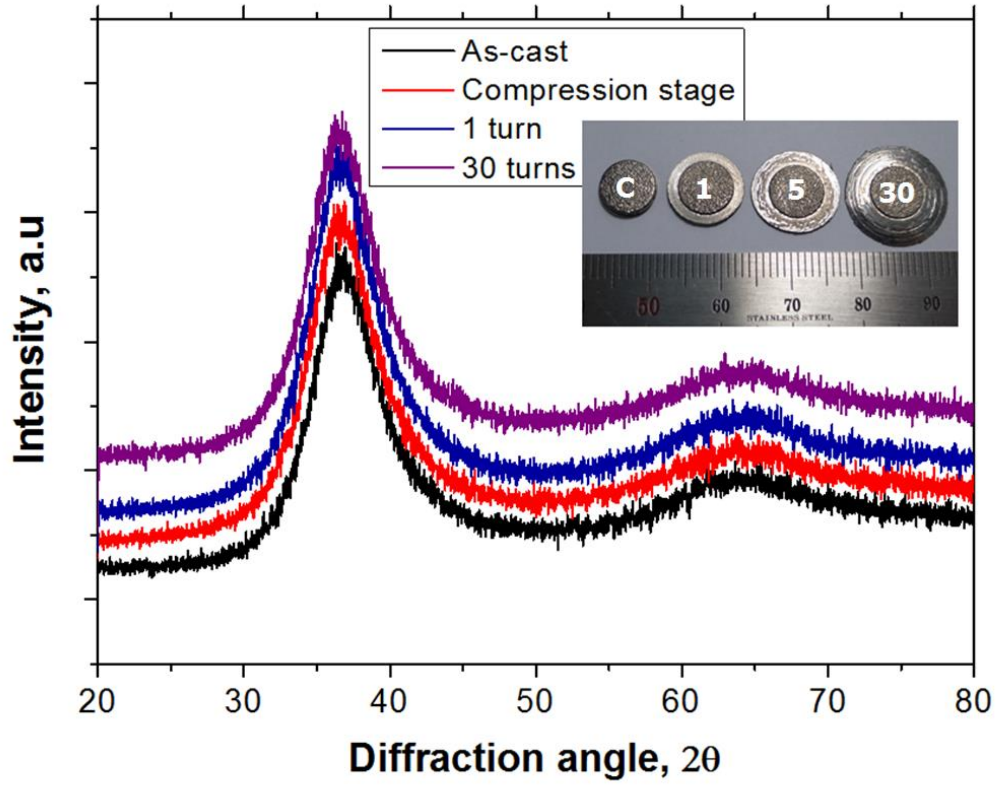

Figure S3. X-ray diffraction patterns of the as-cast and HPT-processed BMGs. The deformed workpieces are shown in the inset.

The XRD results of the as-cast and HPT-processed BMGs are exhibited in Figure S3. The results clearly present amorphous nature without any diffraction peaks maintained after the compression and torsional stages. The inset figure provides the shapes of the deformed workpieces after the compression stage and 1, 5, and 30 turns torsional stages. During the compression stage, the BMG workpiece was slightly deformed at the edge. The workpieces were highly deformed during the torsional stage. In this study, quasi-constrained HPT anvils were used. Consequently, the flash region (outside flow of the material) was not avoidable. When the number of turns increased, the flash region became larger and thickness of the workpiece became thinner. Fracture did not occur until 30 turns of the HPT process.

Specific heat differences

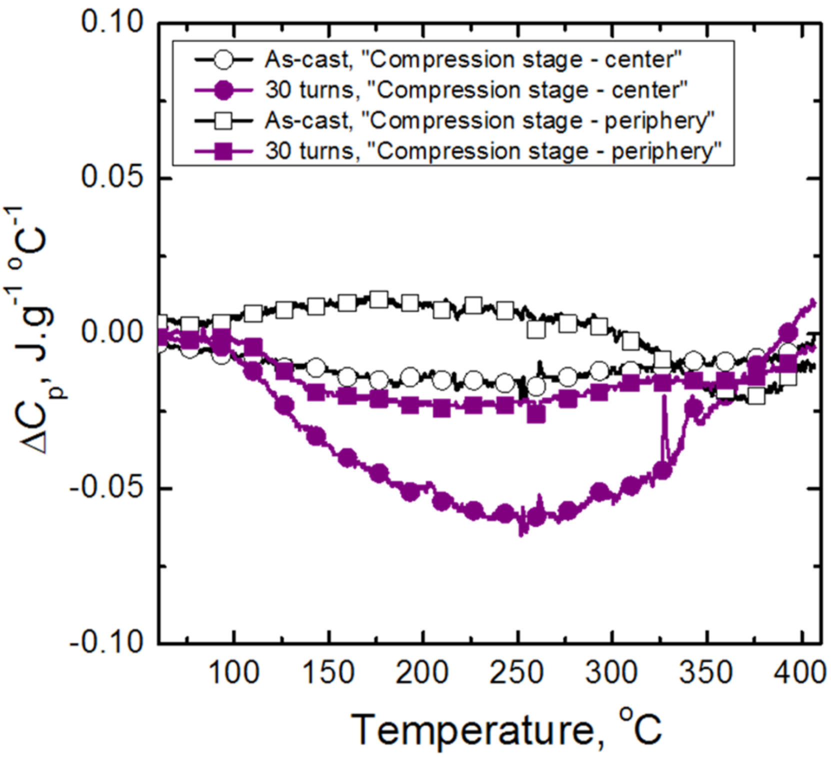

Figure S4. The specific heat differences between compression stage and as-cast samples as well as the difference between compression stage and 30 turn samples are plotted.

The enthalpy change upon structural relaxation is attributable to the change in free volume. Accordingly, plastic strain in the periphery region increases free volume (disordering) during the HPT process. On the other hand, hydrostatic pressure induces free volume annihilation (ordering). Figure S4 exhibits ΔC_p of the as-cast and 30 turns HPT specimens against the compression stage. In the center, disordering seems to occur homogeneously in all activation energy sites as shown in the centers of the as-cast and 30 turns HPT specimens, thus, the shape of ΔC_p against "compression stage – center" does not change so much. Interestingly, disordering seems to occur from both high and low activation energy sites (high and low temperature sites) as shown in the 30 turns HPT sample periphery.

The as-cast specimen presents relatively high C_p at low activation energy sites due to plastic deformation in the compression stage specimen in the periphery region. The negative ΔC_p against pressure in the periphery of the as-cast sample is due to different initial free volume changes of T_g onset of each specimens in DSC scans.

Details of fractured tensile specimens

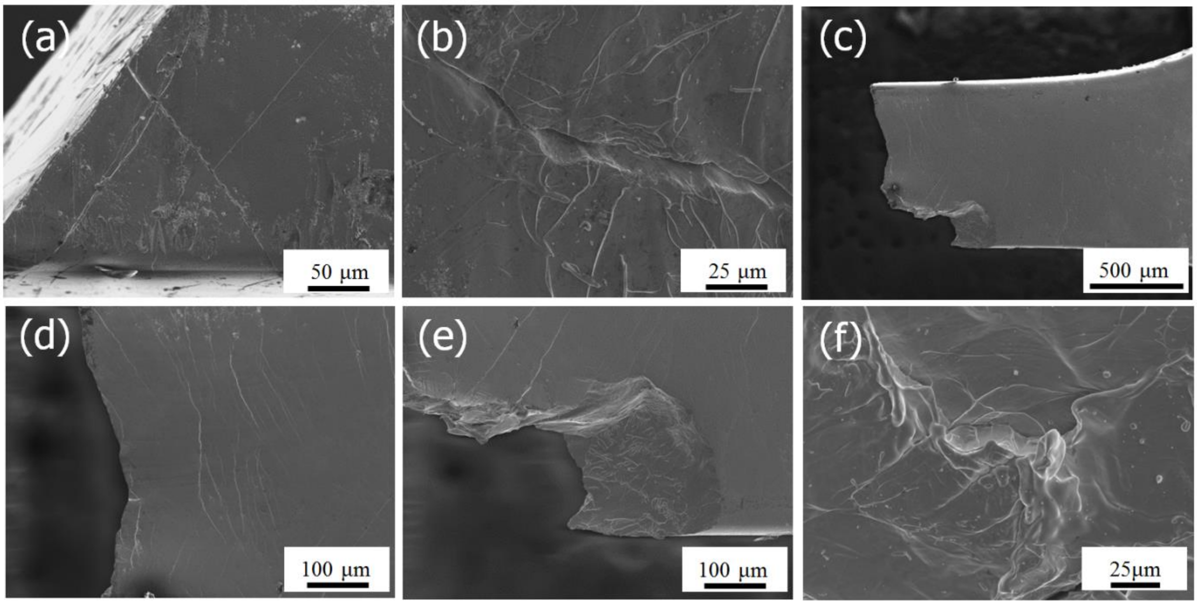

Figure S5. SEM images of (a) lateral surface of the compression-stage specimen after a tensile test, (b) sheared fracture surface due to a link between fracture surface and another shear plane of the compression-stage specimen, (c) lateral surface of the 5 turn specimen, (d) shear bands of the 5 turn specimen, (e) fractured surface parallel to the thickness direction of the 5 turn disk specimen, and (f) rugged facture surface of the 5 turn specimen.

In the compression-stage HPT specimen, Figure S5(a), a few minor shear bands were found around the fracture surface. There were interactions between shear bands. Even though plastic strain was confined in the fractured major shear band, minor shear bands were attributed to small plastic strain. The tensile specimens of the as-cast and compression-stage

HPT samples were fractured straightly through the tensile fracture angle, θ_T, which were both about 55°.

In contrast, the fracture angles of the 5 turn HPT specimens, Figure S5(c), were not straight and did not exhibit the typical tensile fracture angle. There were many minor shear bands near the fracture surface (Figure S5(d)), and some fractured regions were parallel with the thickness direction of the disk specimen (Figure S5(e)). In the 5 turn specimen, the directions of prominent shear bands were different at the center and periphery regions (Fig. 4). Many prominent shear bands were aligned with the thickness direction. However, the shear band structure of the 30 turn specimen was uniform over the whole radial cross section. In the tensile results, the 5 turn specimen showed fracture surfaces along the thickness direction of the disk at the center region with lower ductility compared to the 30 turn specimens. The direction of pre-introduced shear bands affected the compressive deformation behavior[1].

In the 30 turns HPT specimen, more shear bands were formed, compared to the 5 turns HPT specimen (Fig. 3(d)). Besides, a big crack was generated during the tensile test, but the crack propagation was stopped by shear band multiplication at the crack tip (Fig. 3(e)). This phenomenon implies that, the BMG has better toughness and reliable tensile properties after 30 turns of the HPT process. The wavy and tangled pre-existing shear bands achieved by the high shear of the HPT process in this study are more beneficial than pre-existing shear bands aligned with the thickness direction. In the Zr-based BMG of this study, approximately 30 turns is necessary to achieve the uniform shear band structure from the center to edge regions

Fracture of the as-cast specimen resulted from a single shear-off plane, and the fracture surface had a combined feature of vein and cores, as can be seen in Figs. 3(a) and 3(b). The compression stage specimen had similar features to the as-cast specimen. However, there were links between the fracture surface and other shear planes (Figure S5(b)). The

serrated flow in a stress-strain curve of the compression-stage HPT specimen is related to not only the major shear band but also some minor shear bands. The shear band interactions should have beneficial effects on tensile ductility.

After the torsional-stage HPT, fracture surfaces were not straight but rugged. The detailed image of the rugged pattern in Figure S5(f) signified high fracture resistance of the HPT-processed BMG.

Temperature increment during the HPT process

In order to check the deformation heat effect in this study, temperature increment of the BMG during the HPT process was measured. A thermocouple was attached at a fixed upper anvil, and the distance between the thermocouple and the HPT-processed workpiece was 28 mm. The maximum temperature increment after the 30 turns of HPT was 8 K. The anvils have high thermal conductivity and volume, and the contact area between the anvils and the workpiece is large. Therefore, generated heat can be easily diffuse out through the anvils. The increased temperature during the HPT process was much less than the crystallization temperature. The XRD results support that heat generated during the HPT process is not enough for crystallization of the BMG.

Primary phase

After annealing at 450 ℃ for 10 min in a conventional furnace, both as-cast and HPT-BMG did not show any crystallization (Fig. S6(a)). After 20 min at 450 ℃, the diffraction peaks in XRD patterns of the as-cast and HPT processed BMG were both identified as the icosahedral phase (Fig. S6(a)). Since other crystalline peaks are not reflected in both XRD patterns, the primary phase is equally a single icosahedral phase.

Then, we annealed as-cast and BMG-HPT samples at a faster heating condition because structural relaxation occurred before T_x and the microstructural changes obtained by the HPT could not have no effect on crystallization in the conventional furnace. A dilatometer machine (THETA: Transformation Analysis System) was used, and s-type thermocouple directly detected the heating rate of specimens. The measured heating rate was 80 ℃/sec. The icosahedral phase was still the primary phase of the both as-cast and HPT-BMG (Fig. S6(b)). The kinetics of crystallization process is linked to the initial size distribution and density of the nuclei in the amorphous matrix[2,3]. The $Zr_{65}Al_{7.5}Ni_{10}Cu_{12.5}Pd_5$ alloy does not show any nanocrystalline after the HPT in this study, and the primary phase of the HPT-BMG was same with an as-cast material. However, a nanoscale structure arranging fcc Zr_2Ni nanocrystalline particles in a band-like formation in the glassy matrix was easily observed after a compression test in Pd added Zr-based alloys[4,5]. These results imply nanocrystal does not exist in the HPT-BMG because large hydrostatic pressure prevents phase transformation of this alloy.

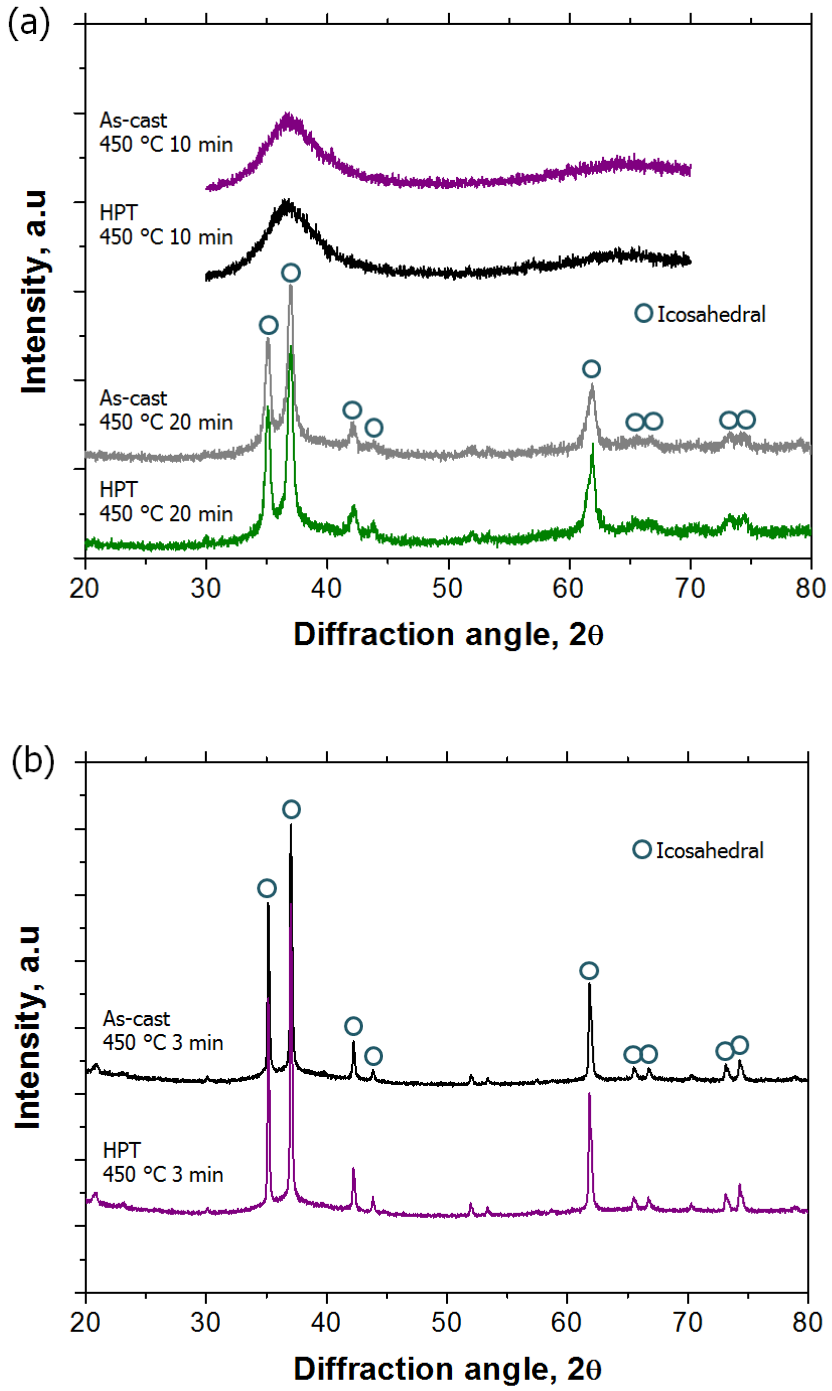

Figure S6. X-ray diffraction patterns of the as-cast and HPT-processed BMGs annealed by (a) a conventional furnace, (b) a dilatometer machine.

References

1. Kobata, J. *et al*. Effect of Pre-Introduced Shear Bands Direction on Deformation Behavior in $Zr_{55}Al_{10}Ni_5Cu_{30}$ Bulk Metallic Glass. *Mater. Trans.* **50**, 2355-2358 (2009).
2. Perepezko, J.H. & Hebert, R.J. Amorphous aluminum alloys-synthesis and stability. *JOM.* **54**, 34-39 (2002).
3. Perepezko, J.H. *et al*. Nanocrystallization reactions in amorphous aluminum alloys. *Mater. Trans.* **44**, 1982-1992 (2003).
4. Saida, J., Setyawan, A.D., Kato, H., Matsushita M. & Inoue, A. Improvement of plasticity in Pd containing Zr-Al-Ni-Cu bulk metallic glass by deformation-induced nano structure change. *Mater. Trans.* **49**, 2732-2736 (2008).
5. Saida, J., Setyawan, A. D. H., Kato, H. & Inoue, A. Nanoscale multistep shear band formation by deformation-induced nanocrystallization in Zr-Al-Ni-Pd bulk metallic glass. *Appl. Phys. Lett.* **87**, 151907 (2005).
